# ER Stress and Lipid Metabolism in Adipocytes

**DOI:** 10.1155/2012/312943

**Published:** 2012-02-12

**Authors:** Beth S. Zha, Huiping Zhou

**Affiliations:** ^1^Department of Microbiology and Immunology, School of Medicine, Virginia Commonwealth University, 1217 East Marshall Street, MSB no. 533, Richmond, VA 23298, USA; ^2^Department of Internal Medicine, McGuire Veterans Affairs Medical Center, Richmond, VA 23298, USA

## Abstract

The role of endoplasmic reticulum (ER) stress is a rapidly emerging field of interest in the pathogenesis of metabolic diseases. Recent studies have shown that chronic activation of ER stress is closely linked to dysregulation of lipid metabolism in several metabolically important cells including hepatocytes, macrophages, *β*-cells, and adipocytes. Adipocytes are one of the major cell types involved in the pathogenesis of the metabolic syndrome. Recent advances in dissecting the cellular and molecular mechanisms involved in the regulation of adipogenesis and lipid metabolism indicate that activation of ER stress plays a central role in regulating adipocyte function. In this paper, we discuss the current understanding of the potential role of ER stress in lipid metabolism in adipocytes. In addition, we touch upon the interaction of ER stress and autophagy as well as inflammation. Inhibition of ER stress has the potential of decreasing the pathology in adipose tissue that is seen with energy overbalance.

## 1. Introduction

In the last two decades, the complexity of adipose tissue has finally become apparent. Investigations surrounding the biological impact of obesity, insulin resistance, and the metabolic syndrome have surged, resulting in a more intricate understanding of “fat.” Adipose tissue (AT) is not only highly specialized to store long-term energy, but is also a central endocrine organ. Therefore, AT is inherently involved in the interplay of inflammatory cascades and energy metabolism, which are important players in metabolic disorders. Even more, sick fat, or adiposopathy, has now been coined an independent endocrine disease [1]. 

Adiposopathy can occur environmentally through over-nutrition. Adipocytes store extra energy in the form of triglycerides (TG) inside cytosolic organelles (lipid droplets, or LD). When there is a continuous need to store TGs, adipocytes must expand in size while continuously being stressed to synthesize more proteins for LD formation. There is an inherent threshold at which adipocytes become too stressed, secrete multiple cytokines, and can no longer expand. The cytokines released activate resident macrophages and call in circulating macrophages, which begin to attempt to engulf these cells, forming the signature “crownlike structures” found in obese tissue [2]. 

During this cascade, increased cytokines can increase adipocyte lipolysis. Increased lipolysis leads to an increase of circulating free fatty acids (FFA) that are deposited in muscle and liver (“lipid dumping”) and results in a decreased insulin sensitivity in these tissues (reviewed in [3]). Particularly, FFA from visceral AT is directly deposited into the portal vein, increasing the risk of fatty liver disease. This may be the underlying basis of current clinical understanding that increased visceral fat is a high-risk factor for cardiovascular disease [4, 5]. 

An increase in FFA release is not only induced by an inflammatory state in AT, but also cellular insulin insensitivity. For this reason, most literature focusing on adipocyte dysregulation in metabolic disease concentrates on the nutrient sensing pathways. However, another important pathway involved in adipocyte pathology is the induction of endoplasmic reticulum (ER) stress. In the past, overstimulation of ER stress has been linked to diseases of genetics and aging (reviewed in [6]), but may in fact be involved in more environmentally induced diseases as well. This paper discusses the recent understanding regarding the role of ER stress in regulating lipid metabolism in adipocytes and the clinical consequence therein.

## 2. ER Stress in the Adipocyte

Numerous cellular pathways can be altered in times of stress, leading to cellular aberrations and dysfunction. However, in the realm of overnutrition, ER stress is arguably the most common and important [7–10]. The ER is central for protein folding, secretions (e.g., cytokines), calcium homeostasis, and lipid synthesis. In the adipocyte, the ER is directly involved with LD formations and maintenance of lipid homeostasis.

Inducing ER stress is relatively effortless *via* depletion of ER calcium stores, changes in ER lipid membrane composition, reactive oxygen species (ROS), or accumulation of misfolded and/or unfolded proteins. When triggered, the ER signals to the cell through the unfolded protein response (UPR) to aid in increased production of proteins needed for protein folding, while decreasing transcription and increasing degradation of other nonessential proteins. If the UPR is unable to return the ER to homeostatic conditions, it will trigger apoptosis.

A central component of the UPR is an ER chaperone protein, BiP/GRP78. In homeostatic conditions, BiP/GRP78 is bound to three ER membrane resident proteins. An insult that alters ATP in the lumen decreases calcium, or increases a demand for protein folding causes GRP78 to unbind. These three proteins, ER transmembrane kinase/endoribonuclease **IRE1**, double-stranded RNA-activated protein kinase-like ER kinase (**PERK**), and activating transcription factor 6 (**ATF-6**), trigger a cascade upon their release, which ultimately leads to the activation of transcription factors that upregulate protein chaperones, proteasome components, and with continuous activation, turns on GADD-153/**CHOP **(C/EBP homologous protein), a major transcriptional factor responsible for ER-stress-induced apoptosis.

### 2.1. IRE1

Upon release from GRP78, IRE1 transautophosphorylates, activating its RNase activity. The activated IRE1 specifically acts on its downstream target X-box-binding protein 1 (**XBP1**) and removes a 26 base pair intron sequence of XBP1 resulting in the formation of spliced XBP1 (XBP1^s^). There are multiple targets of XBP1^s^, such as ER protein chaperones and proteins involved in ER-associated degradation (ERAD) [11–13]. However, beyond the traditional genes it activates, the biological function of XBP1^s^ has now been shown to be more diverse.

In fact, XBP1's ability to induce many ER proteins, and increase expansion of the rough ER [14] has demonstrated its necessity in ER biogenesis. Specific and elaborate knockout models have demonstrated this further; when the ER was poorly developed, secretory cells subsequently failed to function [15, 16]. Sriburi et al. have found that overexpression of XBP1^s^ in preadipocytes induces upregulation of the rate-limiting enzyme in phosphatidylcholine synthesis (CTP: phosphocholine cytidylyltransferase or CCT) [14, 17]. As this is the major phospholipid found in the ER membrane, it follows that XBP1 increases ER biogenesis by both stimulation of ER proteins and membrane components.

This activity of XBP1 is most likely not cell specific, due to the already described centrality of this transcription factor in secretory cell types and hepatocytes. What is of interest in adipocytes, however, is the close interplay of ER biogenesis and LD formations. LDs, as mentioned previously, are a central organelle in adipocytes, though they also are found to a much lesser extent in other cells such as hepatocytes and macrophages. LDs are known to contain a core of triacylglycerols and cholesterol, but the multiple proteins found in their phospholipid monolayer are only beginning to be understood [18]. Although it is already known that the ER assembles and processes the lipids and proteins needed for LD formation, it is not fully known how they are transferred. The formation of a naïve LD is hypothesized to occur when neutral lipids accumulate at the ER membrane and then bud off. However, others propose LDs form as a bicelle or vesicular budding. In addition, the ER may in fact remain linked to LDs, allowing free exchange of proteins [19, 20].

Beyond the debate on whether these two organelles are physically linked, there is no dispute on the centrality of CCT. When CCT is limited, LDs begin to fuse due to less phosphatidylcholine on their surface [21]. Even more, when one gene of CCT was knocked down 60% in drosophila, there was a significant increase of triacylglycerol content [21]. This may be a compensation in which diacylglycerols normally utilized in the CCT pathway are now channeled to neutral lipids in the LDs. Nonetheless, the main end is larger and denser LDs with less active CCT.

The link between CCT, LDs, and the UPR is most likely the foundation of the essential nature of the IRE1-XBP1 pathway in adipogenesis. XBP1-shRNA-treated preadipocytes fail to differentiate, and only transduction of the XBP1^s^ rescued cells [22]. *In vivo* mouse models are more difficult to handle, as the full XBP1 knockout die *in utero* [15]. To circumvent this, one group has placed a liver-specific XBP1 gene into this model, but even these mice die during the neonatal starvation period [16]. These mice are smaller with a negligible white adipose mass, even compared to their heterozygous counterparts.

The mechanism underlying XBP1's significant role may be due to the upregulation of CCAAT/enhancer-binding protein-*α* (C/EBP*α*) [22]. CCAAT/enhancer-binding proteins are essential transcription factors in adipogenesis, with *β* and *δ* being major players in early differentiation and *α* essential in mid- to late differentiation. Sha et al. found that XBP1^s^ upregulates C/EBP*α*, and C/EBP*β* increases transcription of XBP1 [22]. Therefore, XBP1 is integral in the loop of transcriptional activation of adipocyte differentiation as well as the functional maturation of LD formation.

### 2.2. PERK

The PERK-eIF2*α* pathway is another UPR leg involved in adipogenesis. When released, PERK transautophosphorylates leading to activation of its kinase domain. The major result of this is phosphorylation of eukaryotic translation initiation factor 2*α* (eIF2*α*). In the phosphorylated state, this essential component of the translational machinery cannot recycle GTP, inhibiting general translation but at the same time increasing the translation of mRNAs which contain internal ribosome entry sites, such as ATF-4, BiP/GRP78, and SREBP-1 [23–25].

Activating transcription factor (**ATF**)-4 is a well-studied protein involved in the UPR (reviewed in [26]). This transcription factor is heavily involved in increasing amino acid metabolism and protein transport [27, 28]. Importantly, ATF-4 also upregulates stress-related transcription factors ATF-3 and CHOP. CHOP is a central transcription factor involved in cellular perturbations, including inhibition of adipocyte differentiation [29–31], and ultimately inducing apoptosis. However, there is still necessity of balance as although high induction of ATF-4 will lead to CHOP activation, complete absence will affect AT lipogenesis [32]. More studies are needed to fully understand the role of ATF-4 in lipogenesis in adipocytes.

In contrast, more is understood about **SREBP**s (sterol regulatory element-binding proteins). SREBPs are additional transcription factors found in the ER membrane. There are three isoforms- SREBP-1a, -1c, and -2. SREBP-1c is involved in fatty acid synthesis and lipogenesis, -2 in cholesterol synthesis, and -1a in both pathways. The SREBPs are retained in the ER *via* insulin-induced gene (Insig) binding to SREBP-cleavage-activating-protein-(SCAP-) bound SREBP. At times of sensed decreases in cholesterol or fatty acids, SCAP-SREBP dissociates from Insig and relocates to the Golgi where SREBP is cleaved by two site proteases (S1P and S2P). The mature form of SREBP further translocates to the nucleus, activating genes involved in cholesterol and lipid metabolism, such as 3-hydroxy-3-methylgutaryl-CoA (HMG-CoA) synthase, HMG-CoA reductase, squalene synthase, acetyl-CoA carboxylase (ACC), and fatty acid synthase (FAS). Therefore, disruption of ER homeostasis not only alters protein production, but also affects cholesterol and fatty acid synthesis.

Normally, SREBPs are released when there is a sense of depletion of cholesterol or lipids in the ER membrane. However, SREBP1 processing is also regulated through PERK-eIF2*α*. In fact, knockout of PERK substantially decreases active SREBP1 in mammary glands [33]. This is most likely a result from the recent finding that SREBP1 contains an internal ribosome entry site [23]. Therefore, activation of ER stress will redundantly lead to active SREBP1 through both upregulation of translation and release of protein from the membrane.

 In adipocytes of the SREBPs isoform, -1c is the most highly expressed. SREBP1c is an essential transcription factor during adipogenesis (and thus has a dual name of adipocyte determination and differentiation 1/ADD1). Likewise, the PERK pathway has also been found to be important during differentiation of adipocytes *in vitro *[33]. Overexpression of ADD1/SREBP1c leads to an increase of LD formation in preadipocytes, while conditional overexpression in mouse AT inhibits normal mass growth [34]. In addition, SREBP1c has been shown to directly activate C/EBP*β* [35], further supporting its role in adipogenesis. The contradictory results demonstrated with the above mouse models may demonstrate the balance needed by all transcription factors for functional and normal AT.

### 2.3. ATF-6

There are two genes encoding ATF-6, *α*, and *β*. The *α* isoform is a strong transcriptional activator [36], and the form classically studied during UPR activation. When ATF-6 is released from GRP78, it is translocated to the Golgi *via* a localization signal that was hidden when in the bound form. In the Golgi, ATF-6 is cleaved by the same proteases that process SREBPs, releasing the active cytoplasmic domain, which is a transcription factor. Here, ATF6*α* heterodimerizes with XBP1 and upregulates genes with the ER stress response element (ERSE) in their promoters, including GRP78 [37] and other ER chaperone proteins, CHOP, and even XBP1 (reviewed in [38]).

 In the realm of UPR activation altering lipid metabolism in adipocytes, not much has been noted in the literature concerning ATF6. Knockout mouse models of either ATF6*α* or *β* do not show any striking physiological changes, but have allowed for the clarification that ATF6*α* is the more essential isoform for the ER stress pathway [39], though *β* is also involved [36]. Some work has recently demonstrated that ATF6 activation plays a role in the liver to control lipid deposition [40, 41] through inhibition of SREBP-2 [42]. What is of more importance in the adipocyte is the direct function of ATF6 to upregulate XBP1, described above as central in adipogenesis. 

ATF6*α* heterodimerizes with XBP1^s^ in the nucleus to activate genes downstream of UPR activation. However, it is currently not shown if this relationship is also required for upregulation of C/EBP*α*, or CCT activity. More investigations are needed to completely elucidate the direct function of ATF6 in adipocyte lipid metabolism.

## 3. Autophagy, the UPR, and Lipid Metabolism Dysregulation

Autophagy is a self-protective cellular pathway activated by multiple stimuli including viral infection, perceived starvation, organelle dysfunction, and ER stress (discussed below). However, just as in the case of UPR, autophagy has the ability to increase cellular damage or cell death when overstimulated. The multifaceted autophagic pathway is continuously being studied, as is the capacity of this process to help regulate metabolism in mammalian cells. In the past few years, an expanding area of research has unfurled around autophagy and lipid metabolism regulation. In hepatocytes, autophagosomes aid in the control of lipid accumulations by delivering LDs to lysosomes [43]. Similarly, in neurons altered autophagy leads to lipid accumulation [44]. Due to its obvious role in lipid metabolism, Singh and colleagues have now coined this leg of autophagy as lipophagy, in which lipid droplets are degraded through autophagy rather than lipolysis [45].

Further, components of the autophagosome may be necessary for lipid droplet formations [46]. This link was found through the microtubule-associated protein 1A/1B light chain 3 (**LC3**), an essential protein in the autophagy pathway. At induction of autophagy, a double membrane sequesters components of the cytoplasm through the coordination of multiple proteins and membrane expansion. During the initial stages, cytosolic LC3-I is activated through other autophagic-specific proteins by cleavage and lipidation, converting it to membrane-bound LC3-II. Shibata et al. have found that LC3-II does not only colocalize to autophagosomes (the specific autophagy sequestering vacuoles), but also to LDs in hepatocytes and cardiac myocytes [46]. This same group has also demonstrated that LC3 colocalizes to LDs in differentiating adipocytes by using LC3-siRNA [47]. The siRNA of LC3 drastically decreased the ability of adipogenesis [47]. LC3-II has been shown to have tethering capacity to help the fusion of autophagosomes to lysosomes [48]. Therefore, there is a hypothesis that LC3-II is acting to bring LDs into the autophagosome pathway for downstream lipid breakdown [43, 49]. This would provide another pathway of lipid flux beyond lipases acting directly on the LD.

Knockout models have demonstrated how essential autophagy is in adipogenesis. Baerga et al. were able to establish this by first showing the significant increase of autophagosome formations during induction of adipogenesis, followed by the inhibition of differentiation in a knockout *atg5 *mouse model [50]. *Atg5 *encodes a protein that is required similarly to LC3 for the maturation of the pre-autophagosome. Using this model, Baerga et al. saw both *in vitro *and *in vivo *that inhibition of autophagy restrained maturation of preadipocytes, resulting in a marked reduction of WAT in neonatal mice (this mouse model is not able to survive the neonatal starvation period). Of most interest, in the knockout mouse embryonic fibroblasts induced to differentiate, cells that began to mature died through apoptosis, while those in the same culture that did not begin to differentiate remained alive. This study was followed by another with adipose-specific deletion of *atg7 *[51], the gene encoding an essential protein upstream of Atg5. Interestingly, WAT tissue of this knockout model was more characteristic of BAT in both morphology (smaller cells and LDs) and enzyme levels. The importance of Atg7 in adipogenesis was confirmed by Singh et al. who knocked down the same gene, but used slightly different cell lines and mouse model [43]. However, both groups came upon the same finding that the autophagic pathway is essential in adipogenesis.

The trigger of autophagy activation during adipogenesis is currently not known. However, PPAR*γ*, an essential transcription factor of adipogenesis, may be involved. In one cancer cell line, it was found that PPAR*γ* agonists can activate the autophagy pathway [52]. Yet, there is another study that contradicts these findings [53], and such investigations have not yet been repeated in an adipocyte model. Nonetheless, the summation of above experiments does demonstrate that autophagy is essential in adipogenesis, and without, may cause a transdifferentiation of WAT to BAT. On the other hand, a decrease of autophagy in the liver leads to lipid overload in hepatocytes. Intuitively, the difference lies in the biology of the two cell types, where adipocytes are normally storing lipids and hepatocytes are not. In metabolic disease states, such as the metabolic syndrome, it is easy to conceive how dysregulation of autophagy could ultimately lead to fatty liver with increased TG storage in the liver and decreased storage in AT.

## 4. Autophagy and ER Stress

Autophagy and ER stress pathways are not disconnected from one another as previously assumed. In contrast, activation of both can aid in cell survival at times of stress. For one example, autophagy offers an alternative pathway for degradation of proteins when ER-activated proteasomes can no longer handle the load [54–58]. In addition, activation of cell death of each pathway may be interlinked. While classic knowledge is based on ER stress activating apoptosis through CHOP upregulation and autophagy-mediated cell death via a completely separate process, recent findings demonstrate that these two cell death pathways are interlinked.

In more noxious circumstances, it has been shown that cell death through prolonged UPR activation can occur through autophagy-induced cell death [55]. Likewise, inhibition of autophagy increases cell viability with prolonged ER stress [59–61]. However, in nutrient overload and metabolic disorders, impaired autophagy can increase ER stress [62], perhaps due to decreased aberrant protein degradation and energy turnover needed to maintain ER homeostasis. This complex crosstalk of ER stress with the autophagy pathway is not yet well understood. Recently, it was found that ER stress activation can inhibit Akt phosphorylation, the upstream inducer of autophagy at times of perceived starvation [63]. However, the responsible protein(s) are still not known and may even be cell-type specific [64].

Another link is hypothesized to occur through the PERK pathway of the UPR [65, 66]. Some studies have shown that PERK phosphorylation of eIF2*α* leads to an upregulation of LC3 [58]. Yet, it has not been shown if this is directly from eIF2*α* phosphorylation inducing LC3 translation, or through ATF-4 activation increasing *Atg12* transcription [67, 68]. In fact, our current studies suggest that HIV Protease inhibitor (PI)-induced activation of autophagy is closely linked to ER stress *via* the ATF-4 pathway. We have found that those HIV PIs that induce metabolic side effects in the clinic also induce ER stress and autophagy in hepatocytes and adipocytes. The corresponding activation of autophagy seems to be one of the underlying factors by which HIV PIs induce dysregulation of lipid metabolism.

**Figure 1:**
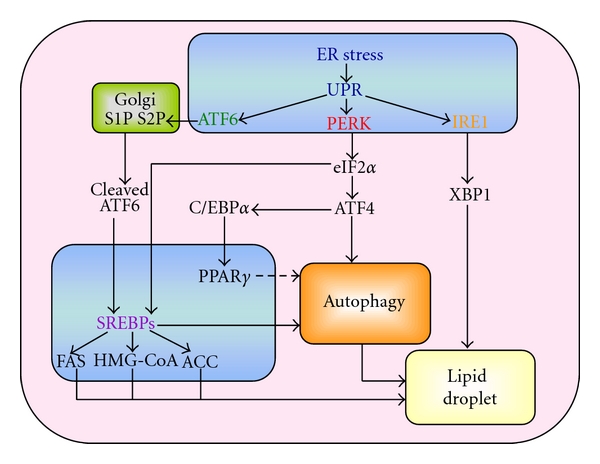
Potential link between ER stress signaling pathways and lipid droplet formation in adipocytes.

 Recent studies have shown a strong link between activation of ER stress, increased autophagy induction, and increased SREBP activity leading to lipid overload in hepatocytes [69], although a mechanism remains to be determined. One group of investigators has demonstrated the capability of SREBP-2 to directly upregulate the expression of autophagy essential proteins [70], giving significance to a previous finding that cholesterol depletion leads to autophagy induction in multiple cell lines [71]. Additionally, knockdown of SREBP-2 decreased LC3 association with LDs in hepatocytes [70]. Although SREBPs are not a current forefront of proposed activators of autophagy, it is probable that in times of cellular lipid depletion, LDs are processed for more essential cellular requirements, and this pathway can be activated through ER stress-induced activation of SREBPs. Although these investigations have not been completed in adipocytes, our laboratory has found that in addition to HIV PIs inducing ER-stress and autophagy in adipocytes, SREBP-1c activation is also altered. Until more investigations are completed, the exact stream can only be hypothesized (Figure 1).

## 5. ER Stress and Inflammation

Obesity and resulting metabolic diseases such as insulin resistance are now known to be strongly associated with chronic inflammation, a substantial risk factor for further complications, most notably atherosclerosis. Increased plasma concentrations of IL-6 and TNF-*α* have been repeatedly noted in obese individuals [72–74]. Investigations into mechanisms underlying obesity and diabetes has demonstrated that inflammation in AT can detrimentally alter human physiology.

With increasing overload, adipocytes begin to hypertrophy. Cells become stressed from the actual expansion and from exceeding an adequate oxygen diffusion distance in tissue [75, 76]. Adipocytes then signal with a release of proinflammatory IL-6 and TNF-*α* cytokines, which activate resident macrophages as well as induce infiltration of circulating macrophages. Stressed adipocytes are subsequently engulfed, resulting in the formation of characteristic crown-like structures.

During this process, released IL-6 and TNF-*α* from stressed adipocytes and activated macrophages can inhibit adipogenesis [77]. In fact, TNF-*α* alone is enough to inhibit induction of PPAR*γ* and C/EBP*α* [78]. Even more, the induction of inflammation can also lead to insulin resistance in AT, already well known and continuously investigated [79–82]. Taken together, the ability to store excess energy in AT is drastically decreased with the decrease of mature adipocytes and the death of cells.

Even more, ER stress has been shown to be activated at times of overnutrition [8]. In adipocytes, ER stress can be activated due to the need of LD synthesis, enzyme production, and conversion of energy to TG at times of overnutrition. Importantly, ER stress has repeatedly been shown to induce the cellular inflammatory cascade through the c-Jun N-terminal kinase (**JNK**) pathway, and JNK has been shown to be upregulated in AT of obese individuals [83, 84]. Additionally, ER stress may trigger the adipocyte inflammatory cascade through PERK activating IKB kinase *β* (**IKK** **β**) when cells are stimulated with free fatty acids [85]. This pathway is also known to be a heavy regulator of inflammatory cytokine release and, together with JNK activation, would lead to the proinflammatory state seen in AT in metabolic disease states.

**Figure 2:**
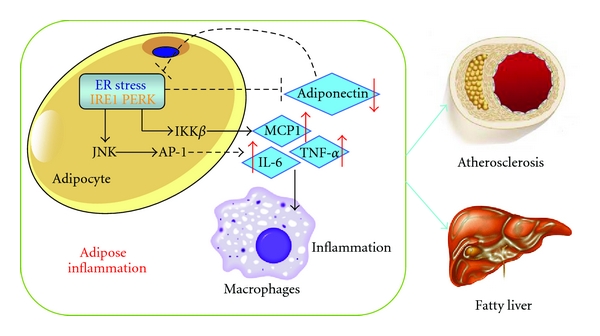
ER-stress-induced inflammation in adipocytes and macrophages contributes to atherosclerosis and fatty liver diseases.

Proinflammatory profile at times of overnutrition is not unique to AT, but occurs throughout the body. However, AT is unique in that it is solely responsible for the subsequent decrease of adiponectin secretion. Adiponectin is an adipocyte-specific anti-inflammatory cytokine that negatively correlates with cardiovascular disease and fatty liver disease [86–88], with a decrease of secretion in overexpanded or stressed tissue [89]. It has been found that adiponectin can alleviate ER stress [90]. Zhou et al. have shown that ER stress initiation is sufficient to decrease adiponectin release. In animal models, they demonstrated that stabilization of adiponectin protein can decrease obesity-induced ER stress in AT [90]. *In vitro*, induction of autophagy could alleviate ER stress responses and subsequently stabilize adiponectin secretions [91]. These are promising findings, and more studies are needed to determine if upregulation of autophagy could ultimately lead to therapeutic options for metabolic diseases (Figure 2).

## 6. Future Directions

We have provided ample references demonstrating that ER stress can induce lipid metabolism dysregulation in adipocytes. Such an assertion is not only important for interested molecular biologists, but for clinicians as well. It has been shown that fat depots of obese patients have increased ER stress [84, 92, 93]. What is more, there may be a link between ER stress upregulation, the inflammatory state of this tissue, and insulin resistance [84, 92, 94, 95].

 The cycle of overnutrition, ER stress, and AT pathology is complex. With the information provided here and our own findings, we support the hypothesis that inhibiting ER stress activation may be therapeutically beneficial in the treatment of metabolic diseases. Chaperones, which enhance ER-protein-folding capacity, have shown potential in the laboratory.

 Two chaperones already FDA approved have been studied in hepatocytes, adipocytes, and *β*-cells for their ability to relieve ER-stress-induced dysfunctions, namely, 4-phenylbutyric acid (PBA) and taurine-conjugated ursodeoxycholic acid (TUDCA). Both were shown to relieve insulin resistance in adipocytes at times of ER stress [96, 97]. In addition, they were able to decrease JNK and IKK*β* activity when cells were stimulated with ER stress inducers, including free fatty acids [85, 96]. *In vivo*, PBA and TUDCA were able to relieve ER stress activation in obese mice [96]. However, further studies are needed to confirm these beneficial effects and elaborate on the extent that chaperone treatment may aid in nutrition overload-induced ER stress and downstream alterations.

 Inhibiting ER stress activation may be the key to an approach for metabolic syndrome therapy. However, more questions remain in this field. Namely, the role of all parts of the UPR in adipocyte lipid metabolism needs to be uncovered, and the mechanism intertwining ER stress and autophagy needs to be further elucidated. Understanding these missing components will allow not only further understanding of key lipid pathways in a central metabolic cell type, but also help determine the best approach that can be utilized for clinical metabolic dysfunctions in patients with altered AT physiology.

## Abbreviations

ACC: Acetyl-CoA carboxylase
Add1: Adipocyte determination and differentiation 1
AT: Adipose tissue
ATF: Activating transcription factor
CHOP: C/EBP homologous protein
CCT: CTP phosphocholine cytidylyltransferase
eIF2*α*: Eukaryotic translation initiation factor
ER: Endoplasmic reticulum
FAS: Fatty acid synthase
FFA: Free fatty acids
Insig: Insulin-induced gene
HMG-CoAR: 3-Hydroxy-3-methylgutaryl-CoA reductase
IKK*β*: IKB kinase *β*
IRE1: Inositol requiring enzyme 1
IRS1: Insulin response substrate
JNK: cJun N-terminal kinase
LC3: Microtubule associated light chain protein
LD: Lipid droplet
PBA: Protein-1 namely 4-phenylbutyric acid
PERK: PKR-like eukaryotic initiation factor 2*α* kinase
SCAP: SREBP cleavage activating protein
SREBP: Sterol regulatory element binding protein
TUDCA: Taurine-conjugated ursodeoxycholic acid
TG: Triglyceride
UPR: Unfolded protein response
XBP1: X-box binding protein.

## References

[1] Bays HE, González-Campoy JM, Henry RR (2008). Is adiposopathy (sick fat) an endocrine disease?. *International Journal of Clinical Practice*.

[2] Surmi BK, Hasty AH (2008). Macrophage infiltration into adipose tissue: initiation, propagation and remodeling. *Future Lipidology*.

[3] Mittra S, Bansal VS, Bhatnagar PK (2008). From a glucocentric to a lipocentric approach towards metabolic syndrome. *Drug Discovery Today*.

[4] Kabir M, Catalano KJ, Ananthnarayan S (2005). Molecular evidence supporting the portal theory: a causative link between visceral adiposity and hepatic insulin resistance. *American Journal of Physiology*.

[5] Yoshii H, Lam TKT, Gupta N (2006). Effects of portal free fatty acid elevation on insulin clearance and hepatic glucose flux. *American Journal of Physiology*.

[6] Yoshida H (2007). ER stress and diseases. *FEBS Journal*.

[7] Alhusaini S, McGee K, Schisano B (2010). Lipopolysaccharide, high glucose and saturated fatty acids induce endoplasmic reticulum stress in cultured primary human adipocytes: salicylate alleviates this stress. *Biochemical and Biophysical Research Communications*.

[8] Boden G, Duan X, Homko C (2008). Increase in endoplasmic reticulum stress-related proteins and genes in adipose tissue of obese, insulin-resistant individuals. *Diabetes*.

[9] Gregor MF, Yang L, Fabbrini E (2009). Endoplasmic reticulum stress is reduced in tissues of obese subjects after weight loss. *Diabetes*.

[10] Sharma NK, Das SK, Mondal AK (2008). Endoplasmic reticulum stress markers are associated with obesity in nondiabetic subjects. *Journal of Clinical Endocrinology and Metabolism*.

[11] Yoshida H, Matsui T, Yamamoto A, Okada T, Mori K (2001). XBP1 mRNA is induced by ATF6 and spliced by IRE1 in response to ER stress to produce a highly active transcription factor. *Cell*.

[12] Yoshida H, Matsui T, Hosokawa N, Kaufman RJ, Nagata K, Mori K (2003). A time-dependent phase shift in the mammalian unfolded protein response. *Developmental Cell*.

[13] Lee AH, Iwakoshi NN, Glimcher LH (2003). XBP-1 regulates a subset of endoplasmic reticulum resident chaperone genes in the unfolded protein response. *Molecular and Cellular Biology*.

[14] Sriburi R, Jackowski S, Mori K, Brewer JW (2004). XBP1: a link between the unfolded protein response, lipid biosynthesis, and biogenesis of the endoplasmic reticulum. *Journal of Cell Biology*.

[15] Reimold AM, Iwakoshi NN, Manis J (2001). Plasma cell differentiation requires the transcription factor XBP-1. *Nature*.

[16] Lee AH, Chu GC, Iwakoshi NN, Glimcher LH (2005). XBP-1 is required for biogenesis of cellular secretory machinery of exocrine glands. *EMBO Journal*.

[17] Sriburi R, Bommiasamy H, Buldak GL (2007). Coordinate regulation of phospholipid biosynthesis and secretory pathway gene expression in XBP-1(S)-induced endoplasmic reticulum biogenesis. *Journal of Biological Chemistry*.

[18] Farese RV, Walther TC (2009). Lipid droplets finally get a little R-E-S-P-E-C-T. *Cell*.

[19] Walther TC, Farese RV (2009). The life of lipid droplets. *Biochimica et Biophysica Acta*.

[20] Robenek H, Buers I, Robenek MJ (2011). Topography of lipid droplet-associated proteins: insights from freeze-fracture replica immunogold labeling. *Journal of Lipids*.

[21] Guo Y, Walther TC, Rao M (2008). Functional genomic screen reveals genes involved in lipid-droplet formation and utilization. *Nature*.

[22] Sha H, He Y, Chen H (2009). The IRE1*α*-XBP1 pathway of the unfolded protein response is required for adipogenesis. *Cell Metabolism*.

[23] Damiano F, Alemanno S, Gnoni GV, Siculella L (2010). Translational control of the sterol-regulatory transcription factor SREBP-1 mRNA in response to serum starvation or ER stress is mediated by an internal ribosome entry site. *Biochemical Journal*.

[24] DeGracia DJ, Kumar R, Owen CR, Krause GS, White BC (2002). Molecular pathways of protein synthesis inhibition during brain reperfusion: implications for neuronal survival or death. *Journal of Cerebral Blood Flow and Metabolism*.

[25] Yang Q, Sarnow P (1997). Location of the internal ribosome entry site in the 5’ non-coding region of the immunoglobulin heavy-chain binding protein (BiP) mRNA: evidence for specific RNA-protein interactions. *Nucleic Acids Research*.

[26] Ameri K, Harris AL (2008). Activating transcription factor 4. *International Journal of Biochemistry and Cell Biology*.

[27] Rutkowski DT, Kaufman RJ (2003). All roads lead to ATF4. *Developmental Cell*.

[28] Wek RC, Jiang HY, Anthony TG (2006). Coping with stress: EIF2 kinases and translational control. *Biochemical Society Transactions*.

[29] Batchvarova N, Wang XZ, Ron D (1995). Inhibition of adipogenesis by the stress-induced protein CHOP (Gadd153). *EMBO Journal*.

[30] Clarke SL, Robinson CE, Gimble JM (1997). CAAT/Enhancer binding proteins directly modulate transcription from the peroxisome proliferator-activated receptor *γ*2 promoter. *Biochemical and Biophysical Research Communications*.

[31] Adelmant G, Gilbert JD, Freytag SO (1998). Human translocation liposarcoma-CCAAT/enhancer binding protein (C/EBP) homologous protein (TLS-CHOP) oncoprotein prevents adipocyte differentiation by directly interfering with C/EBP*β* function. *Journal of Biological Chemistry*.

[32] Wang C, Huang Z, Du Y, Cheng Y, Chen S, Guo F (2010). ATF4 regulates lipid metabolism and thermogenesis. *Cell Research*.

[33] Bobrovnikova-Marjon E, Hatzivassiliou G, Grigoriadou C (2008). PERK-dependent regulation of lipogenesis during mouse mammary gland development and adipocyte differentiation. *Proceedings of the National Academy of Sciences of the United States of America*.

[34] Shimomura I, Hammer RE, Richardson JA (1998). Insulin resistance and diabetes mellitus in transgenic mice expressing nuclear SREBP-1c in adipose tissue: model for congenital generalized lipodystrophy. *Genes and Development*.

[35] Lay SL, Lefrère I, Trautwein C, Dugail I, Krief S (2002). Insulin and sterol-regulatory element-binding protein-1c (SREBP-1c) regulation of gene expression in 3T3-L1 adipocytes: identification of CCAAT/enhancer-binding protein *β* as an SREBP-1c target. *Journal of Biological Chemistry*.

[36] Thuerauf DJ, Marcinko M, Belmont PJ, Glembotski CC (2007). Effects of the isoform-specific characteristics of ATF6*α* and ATF6*β* on endoplasmic reticulum stress response gene expression and cell viability. *Journal of Biological Chemistry*.

[37] Haze K, Yoshida H, Yanagi H, Yura T, Mori K (1999). Mammalian transcription factor ATF6 is synthesized as a transmembrane protein and activated by proteolysis in response to endoplasmic reticulum stress. *Molecular Biology of the Cell*.

[38] Davenport EL, Morgan GJ, Davies FE (2008). Untangling the unfolded protein response. *Cell Cycle*.

[39] Adachi Y, Yamamoto K, Okada T, Yoshida H, Harada A, Mori K (2008). ATF6 is a transcription factor specializing in the regulation of quality control proteins in the endoplasmic reticulum. *Cell Structure and Function*.

[40] Rutkowski DT, Wu J, Back SH (2008). UPR pathways combine to prevent hepatic steatosis caused by ER stress-mediated suppression of transcriptional master regulators. *Developmental Cell*.

[41] Yamamoto K, Takahara K, Oyadomari S (2010). Induction of liver steatosis and lipid droplet formation in ATF6*α*-knockout mice burdened with pharmacological endoplasmic reticulum stress. *Molecular Biology of the Cell*.

[42] Schröder M, Kaufman RJ (2005). ER stress and the unfolded protein response. *Mutation Research*.

[43] Singh R, Kaushik S, Wang Y (2009). Autophagy regulates lipid metabolism. *Nature*.

[44] Martinez-Vicente M, Talloczy Z, Wong E (2010). Cargo recognition failure is responsible for inefficient autophagy in Huntington’s disease. *Nature Neuroscience*.

[45] Singh R (2010). Autophagy and regulation of lipid metabolism. *Results and Problems in Cell Differentiation*.

[46] Shibata M, Yoshimura K, Furuya N (2009). The MAP1-LC3 conjugation system is involved in lipid droplet formation. *Biochemical and Biophysical Research Communications*.

[47] Shibata M, Yoshimura K, Tamura H (2010). LC3, a microtubule-associated protein1A/B light chain3, is involved in cytoplasmic lipid droplet formation. *Biochemical and Biophysical Research Communications*.

[48] Nakatogawa H, Ichimura Y, Ohsumi Y (2007). Atg8, a ubiquitin-like protein required for autophagosome formation, mediates membrane tethering and hemifusion. *Cell*.

[49] Kovsan J, Bashan N, Greenberg AS, Rudich A (2010). Potential role of autophagy in modulation of lipid metabolism. *American Journal of Physiology*.

[50] Baerga R, Zhang Y, Chen PH, Goldman S, Jin S (2009). Targeted deletion of autophagy-related 5 (atg5) impairs adipogenesis in a cellular model and in mice. *Autophagy*.

[51] Zhang Y, Goldman S, Baerga R, Zhao Y, Komatsu M, Jin S (2009). Adipose-specific deletion of autophagy-related gene 7 (atg7) in mice reveals a role in adipogenesis. *Proceedings of the National Academy of Sciences of the United States of America*.

[52] Zhou J, Zhang W, Liang B (2009). PPAR*γ* activation induces autophagy in breast cancer cells. *International Journal of Biochemistry and Cell Biology*.

[53] Yan J, Yang H, Wang G (2010). Autophagy augmented by troglitazone is independent of EGFR transactivation and correlated with AMP-activated protein kinase signaling. *Autophagy*.

[54] Kawakami T, Inagi R, Takano H (2009). Endoplasmic reticulum stress induces autophagy in renal proximal tubular cells. *Nephrology Dialysis Transplantation*.

[55] Yorimitsu T, Nair U, Yang Z, Klionsky DJ (2006). Endoplasmic reticulum stress triggers autophagy. *Journal of Biological Chemistry*.

[56] Ding WX, Yin XM (2008). Sorting, recognition and activation of the misfolded protein degradation pathways through macroautophagy and the proteasome. *Autophagy*.

[57] Ding WX, Ni HM, Gao W (2007). Linking of autophagy to ubiquitin-proteasome system is important for the regulation of endoplasmic reticulum stress and cell viability. *American Journal of Pathology*.

[58] Kouroku Y, Fujita E, Tanida I (2007). ER stress (PERK/eIF2*α* phosphorylation) mediates the polyglutamine-induced LC3 conversion, an essential step for autophagy formation. *Cell Death and Differentiation*.

[59] Price J, Zaidi AK, Bohensky J, Srinivas V, Shapiro IM, Ali H (2010). Akt-1 mediates survival of chondrocytes from endoplasmic reticulum-induced stress. *Journal of Cellular Physiology*.

[60] Qin L, Wang Z, Tao L, Wang Y (2010). ER stress negatively regulates AKT/TSC/mTOR pathway to enhance autophagy. *Autophagy*.

[61] Ogata M, Hino SI, Saito A (2006). Autophagy is activated for cell survival after endoplasmic reticulum stress. *Molecular and Cellular Biology*.

[62] Yang L, Li P, Fu S, Calay ES, Hotamisligil GS (2010). Defective hepatic autophagy in obesity promotes ER stress and causes insulin resistance. *Cell Metabolism*.

[63] Gupta AK, Li B, Cerniglia GJ, Ahmed MS, Hahn SM, Maity A (2007). The HIV protease inhibitor nelfinavir downregulates Akt phosphorylation by inhibiting proteasomal activity and inducing the unfolded protein response. *Neoplasia*.

[64] Schleicher SM, Moretti L, Varki V, Lu B (2010). Progress in the unraveling of the endoplasmic reticulum stress/autophagy pathway and cancer: implications for future therapeutic approaches. *Drug Resistance Updates*.

[65] Jia W, Loria RM, Park MA, Yacoub A, Dent P, Graf MR (2010). The neuro-steroid, 5-androstene 3*β*,17*α* diol; induces endoplasmic reticulum stress and autophagy through PERK/eIF2*α* signaling in malignant glioma cells and transformed fibroblasts. *International Journal of Biochemistry and Cell Biology*.

[66] Kim KW, Moretti L, Mitchell LR, Jung DK, Lu B (2010). Endoplasmic reticulum stress mediates radiation-induced autophagy by perk-eIF2*α* in caspase-3/7-deficient cells. *Oncogene*.

[67] Rzymski T, Milani M, Pike L (2010). Regulation of autophagy by ATF4 in response to severe hypoxia. *Oncogene*.

[68] Milani M, Rzymski T, Mellor HR (2009). The role of ATF4 stabilization and autophagy in resistance of breast cancer cells treated with Bortezomib. *Cancer Research*.

[69] Nishina S, Korenaga M, Hidaka I (2010). Hepatitis C virus protein and iron overload induce hepatic steatosis through the unfolded protein response in mice. *Liver International*.

[70] Seo Y-K, Jeon T-I, Chong HK, Biesinger J, Xie X, Osborne TF (2011). Genome-wide localization of SREBP-2 in hepatic chromatin predicts a role in autophagy. *Cell Metabolism*.

[71] Cheng J, Ohsaki Y, Tauchi-Sato K, Fujita A, Fujimoto T (2006). Cholesterol depletion induces autophagy. *Biochemical and Biophysical Research Communications*.

[72] Van Guilder GP, Hoetzer GL, Greiner JJ, Stauffer BL, DeSouza CA (2006). Influence of metabolic syndrome on biomarkers of oxidative stress and inflammation in obese adults. *Obesity*.

[73] Tsigos C, Kyrou I, Chala E (1999). Circulating tumor necrosis factor alpha concentrations are higher in abdominal versus peripheral obesity. *Metabolism*.

[74] Kern PA, Ranganathan S, Li C, Wood L, Ranganathan G (2001). Adipose tissue tumor necrosis factor and interleukin-6 expression in human obesity and insulin resistance. *American Journal of Physiology*.

[75] O’Rourke RW (2009). Inflammation in obesity-related diseases. *Surgery*.

[76] Goossens GH (2008). The role of adipose tissue dysfunction in the pathogenesis of obesity-related insulin resistance. *Physiology and Behavior*.

[77] Gustafson B, Hammarstedt A, Andersson CX, Smith U (2007). Inflamed adipose tissue: a culprit underlying the metabolic syndrome and atherosclerosis. *Arteriosclerosis, Thrombosis, and Vascular Biology*.

[78] Meng L, Zhou J, Sasano H, Suzuki T, Zeitoun KM, Bulun SE (2001). Tumor necrosis factor *α* and interleukin 11 secreted by malignant breast epithelial cells inhibit adipocyte differentiation by selectively down-regulating CCAAT/enhancer binding protein *α* and peroxisome proliferator-activated receptor *γ*: mechanism of desmoplastic reaction. *Cancer Research*.

[79] Bastard JP, Maachi M, Lagathu C (2006). Recent advances in the relationship between obesity, inflammation, and insulin resistance. *European Cytokine Network*.

[80] Hotamisligil GS (2006). Inflammation and metabolic disorders. *Nature*.

[81] Monteiro R, Azevedo I (2010). Chronic inflammation in obesity and the metabolic syndrome. *Mediators of Inflammation*.

[82] Xu H, Barnes GT, Yang Q (2003). Chronic inflammation in fat plays a crucial role in the development of obesity-related insulin resistance. *Journal of Clinical Investigation*.

[83] Hirosumi J, Tuncman G, Chang L (2002). A central, role for JNK in obesity and insulin resistance. *Nature*.

[84] Boden G, Merali S (2011). Measurement of the increase in endoplasmic reticulum stress-related proteins and genes in adipose tissue of obese, insulin-resistant individuals. *Methods in Enzymology*.

[85] Jiao P, Ma J, Feng B (2011). FFA-induced adipocyte inflammation and insulin resistance: involvement of ER stress and IKK*β* pathways. *Obesity*.

[86] Polyzos SA, Kountouras J, Zavos C, Tsiaousi E (2010). The role of adiponectin in the pathogenesis and treatment of non-alcoholic fatty liver disease. *Diabetes, Obesity and Metabolism*.

[87] Polyzos SA, Kountouras J, Zavos C (2009). Nonalcoholic fatty liver disease: the pathogenetic roles of insulin resistance and adipocytokines. *Current Molecular Medicine*.

[88] Ros E (2009). Nuts and novel biomarkers of cardiovascular disease. *American Journal of Clinical Nutrition*.

[89] Kern PA, Di Gregorio GB, Lu T, Rassouli N, Ranganathan G (2003). Adiponectin expression from human adipose tissue: relation to obesity, insulin resistance, and tumor necrosis factor-*α* expression. *Diabetes*.

[90] Zhou L, Liu M, Zhang J, Chen H, Dong LQ, Liu F (2010). DsbA-L alleviates endoplasmic reticulum stress-induced adiponectin downregulation. *Diabetes*.

[91] Zhou L, Liu F (2010). Autophagy: roles in obesity-induced ER stress and adiponectin downregulation in adipocytes. *Autophagy*.

[92] Kars M, Yang L, Gregor MF (2010). Tauroursodeoxycholic acid may improve liver and muscle but not adipose tissue insulin sensitivity in obese men and women. *Diabetes*.

[93] Miranda M, Escoté X, Ceperuelo-Mallafré V (2010). Relation between human LPIN1, hypoxia and endoplasmic reticulum stress genes in subcutaneous and visceral adipose tissue. *International Journal of Obesity*.

[94] Marsollier N, Ferré P, Foufelle F (2011). Novel insights in the interplay between inflammation and metabolic diseases: a role for the pathogen sensing kinase PKR. *Journal of Hepatology*.

[95] Hotamisligil GS (2008). Inflammation and endoplasmic reticulum stress in obesity and diabetes. *International Journal of Obesity*.

[96] Özcan U, Yilmaz E, Özcan L (2006). Chemical chaperones reduce ER stress and restore glucose homeostasis in a mouse model of type 2 diabetes. *Science*.

[97] Nakatani Y, Kaneto H, Kawamori D (2005). Involvement of endoplasmic reticulum stress in insulin resistance and diabetes. *Journal of Biological Chemistry*.

